# Satellite cells from dystrophic muscle retain regenerative capacity

**DOI:** 10.1016/j.scr.2014.10.007

**Published:** 2015-01

**Authors:** Luisa Boldrin, Peter S. Zammit, Jennifer E. Morgan

**Affiliations:** a University College London, Institute of Child Health, The Dubowitz Neuromuscular Centre, Molecular Neurosciences Section, Developmental Neurosciences Programme, 30 Guilford Street, London WC1N 1EH, United Kingdom; b King's College London, The Randall Division of Cell and Molecular Biophysics, New Hunt's House, London SE1 1UL, United Kingdom

## Abstract

Duchenne muscular dystrophy is an inherited disorder that is characterized by progressive skeletal muscle weakness and wasting, with a failure of muscle maintenance/repair mediated by satellite cells (muscle stem cells). The function of skeletal muscle stem cells resident in dystrophic muscle may be perturbed by being in an increasing pathogenic environment, coupled with constant demands for repairing muscle. To investigate the contribution of satellite cell exhaustion to this process, we tested the functionality of satellite cells isolated from the *mdx* mouse model of Duchenne muscular dystrophy. We found that satellite cells derived from young *mdx* mice contributed efficiently to muscle regeneration within our *in vivo* mouse model. To then test the effects of long-term residence in a dystrophic environment, satellite cells were isolated from aged *mdx* muscle. Surprisingly, they were as functional as those derived from young or aged wild type donors. Removing satellite cells from a dystrophic milieu reveals that their regenerative capacity remains both intact and similar to satellite cells derived from healthy muscle, indicating that the host environment is critical for controlling satellite cell function.

## Highlights

• Grafted *mdx* satellite cells regenerate muscle as well as wild-type satellite cells.
• Aged *mdx* myofibers bear more satellite cells than aged wild type fibers.
• *mdx* satellite cells retain their ability to activate.
• Aged *mdx* satellite cells are robustly regenerative in vivo.

## Introduction

Skeletal muscle maintenance, repair, and regeneration are mediated by skeletal muscle stem cells. Although there are several cell types resident in skeletal muscle that can contribute to these processes under certain circumstances (Dellavalle et al, 2011; Meng et al, 2011), the principal skeletal muscle stem cell is the satellite cell, located underneath the basal lamina of a myofiber (Mauro, 1961; Relaix and Zammit, 2012). Satellite cells are normally mitotically quiescent, but can be activated to produce myoblast progeny that will differentiate to repair muscle. In healthy muscle, repair is normally a remarkably efficient process. However, it is likely that satellite cell function is compromised in muscular dystrophies, inherited disorders in which there is a loss of muscle structure and function, leading to weakness and disability (Emery, 2002; Morgan and Zammit, 2010).

In Duchenne muscular dystrophy (DMD), the *dystrophin (DMD)* gene is mutated, leading to a loss of dystrophin protein. In healthy skeletal muscle, dystrophin is present beneath the basal lamina of muscle myofibers and interacts with other members of the dystrophin-associated protein complex (DAPC) to maintain muscle structure and function. It also has a signaling role, including mechanotransduction of forces and localization of signaling proteins within muscle myofibers (Emery, 2002). The absence of dystrophin renders a myofiber prone to damage by mechanical stress, leading to necrosis. Although muscle regeneration occurs, the regenerated myofibers still lack dystrophin and consequently undergo further cycles of degeneration and regeneration, which eventually completely fails, with the muscle tissue becoming substituted by fibrotic/adipose/connective tissue and unable to generate sufficient force (Webster and Blau, 1990). As dystrophin protein is part of the force transduction apparatus of a muscle fiber, it should not be expressed in satellite cells until after they undergo myogenic differentiation (Hoffman et al, 1987). Thus, the lack of dystrophin in DMD will have only an indirect effect on satellite cell function, as it leads to chronic fiber necrosis and consequent activation, proliferation and then differentiation of nearby satellite cells in an increasing hostile dystrophic microenvironment (Morgan and Zammit 2010).

The *mdx* mouse is a naturally-occurring genetic and biochemical homologue of DMD and has been widely used as an experimental model. Although *mdx* muscles retain their capacity to regenerate throughout life, certain muscle in old *mdx* mouse, including diaphragm (Stedman et al, 1991), soleus and plantaris muscles (Pastoret and Sebille, 1993), accurately model DMD, exhibiting muscle fiber loss and severe pathological features such as fat infiltration and extensive fibrosis (Pastoret and Sebille, 1995; Wineinger et al, 1998).

In DMD, satellite cell function may be indirectly affected, through constant recruitment to muscle repair and regeneration and so their regenerative capacity may become exhausted by the progression of the dystrophy with time. This may then synergise with the increasing hostile microenvironment of the dystrophic muscle to prevent effective repair (Morgan and Zammit 2010). We hypothesize that long-term residence within a dystrophic muscle environment has a deleterious effect on satellite cell function. We therefore tested specifically the regenerative potential of satellite cells derived from the dystrophin-deficient *mdx* mouse model of DMD at different ages.

Satellite cells isolated from young *mdx* mice were transplanted into a permissive host muscle environment (pre-irradiated muscles of *mdx nude* mice) (Boldrin et al, 2012; Boldrin et al, 2009; Collins et al, 2005; Neal et al, 2012). Surprisingly, satellite cells from young *mdx* muscles were able to contribute efficiently to muscle regeneration. We next isolated satellite cells from aged *mdx* mice to test their capacity to regenerate muscle after long-term residence in a dystrophic environment and found that they too were able to regenerate muscle as efficiently as satellite cells derived from young or aged wild type donors. Our data imply that the impaired muscle regeneration observed in this model of DMD arises mainly from the pathological environment, rather than from endogenous defects in the regenerative capacity of satellite cells.

## Materials and methods

### Donor satellite cell preparation and grafting

Mice were bred and experimental procedures were carried out in the Biological Services Unit of Institute of Child Health, University College London, and in the Biological Services Unit of Kings College London, in accordance with the Animals (Scientific Procedures) Act 1986.

Donor mice were obtained by breeding either homozygote 3F-*nlacZ*-2E mice—whose myonuclei express β-gal (Kelly et al, 1995), or heterozygote *Myf5^nlacZ/+^* mice—that have nlacZ encoding nuclear-localizing β-gal targeted to the *Myf5* locus (Tajbakhsh et al, 1996) that identifies the majority of satellite cells (Beauchamp et al, 2000)—with *mdx* and C57Bl/10 mice. Within muscles grafted with satellite cells derived from 3F-*nlacZ*-2E mice, β-gal identifies myonuclei of donor origin, whereas in muscles grafted with satellite cells derived from *Myf5^nlacZ/+^* mice, β-gal marks satellite cells of donor origin in regenerated muscles (Boldrin et al, 2012; Boldrin et al, 2009; Collins et al, 2005).

Satellite cells were isolated from *extensor digitorum longus* (EDL) muscles of young (2–3 months old) or aged (15 months old for *mdx* × *Myf5^nlacZ/+^* and age matched *Myf5^nlacZ/+^* control mice) donor mice as previously described (Boldrin et al, 2009; Collins and Zammit, 2009). Briefly, the EDL muscles were extracted from tendon to tendon and were then digested in 2% collagenase type I (Sigma)/Dulbecco's modified Eagles medium (DMEM; Gibco) at 35 °C for 70 min. Muscles were serially washed to separate muscle fibers from cell contaminants and debris. Myofibers were counted and triturated with a 19-gauge needle, to allow release of satellite cells. Satellite cells were separated from debris and contracted myofibers using a 40 μm cell strainer and the resulting cell suspension centrifuged. Cells were then suspended in the desired volume and placed immediately on ice before injection (Boldrin 2013). Approximately 400 satellite cells were grafted into each pre-irradiated *tibialis anterior* (TA) muscle of 3 week old *mdx* nude mice, as described previously (Boldrin et al, 2009).

### In vivo assay of donor satellite cell functionality

To investigate functionality of grafted satellite cells, host TA muscles were injected with 10 μl of *notechis scutatus* notexin (10 μg/ml) (Latoxan, France) (Harris, 2003) three weeks after donor cell grafting. Muscles were analyzed a week after notexin injection (Boldrin et al, 2012; Boldrin et al, 2009; Collins et al, 2005; Gross and Morgan, 1999).

### Analysis of grafted muscles

TA muscles were analyzed 4 weeks after grafting (Boldrin et al, 2009). Some muscles grafted with *mdx* × *Myf5^nlacZ/+^* satellite cells derived from young donor mice (Fig. 2) were fixed in 4% paraformaldehyde for 15 min and then X-gal stained to capture images of the whole TA muscles. All grafted muscles were mounted in gum tradacanth and frozen in isopentane chilled in liquid nitrogen for cryosectioning. Serial transverse cryosections were collected at 100 μm intervals throughout the muscle. In muscles grafted with donor satellite cells derived from wild type, rather than *mdx* mice, sections serial to those containing X-gal +ve myonuclei (indicating myofibers of donor origin) were immunostained for dystrophin (P7 antibody, (Lu et al, 2005)) and those with the highest number of dystrophin positive myofibers were used for quantification of donor-derived myofibers. In muscles grafted with satellite cells derived from dystrophin-deficient *mdx* donor mice, quantification of donor-derived myofibers was performed by counting myofibers containing at least one X-gal +ve nucleus.

In grafted muscles that were injected with notexin, neonatal-myosin (BF34 antibody, DSHB) immunostaining was performed in combination with dystrophin staining (Boldrin et al, 2012; Boldrin et al, 2009; Gross and Morgan, 1999).

When counting the number of donor-derived satellite cells in muscles injected with satellite cells expressing *Myf5^nlacZ/+^*, X-gal staining and immunostaining with laminin antibody (Sigma) were performed on the same sections, in order to identify satellite cells located underneath the basal lamina.

### Single fiber immunohistochemistry

Single myofibers isolated from *mdx* × 3F-*nlacZ*-2E and *mdx* × *Myf5^nlacZ/+^* donor mice were stained with X-gal and DAPI to determine expression of the reporter gene. To investigate the phenotype of *Myf5^nlacZ/+^* satellite cells derived from old *mdx* × *Myf5^nlacZ/+^* and *Myf5^nlacZ/+^* mice, single EDL myofibers were isolated from 19 month old *mdx* × *Myf5^nlacZ/+^* male mice, *n* = 3) and a 19 month old *Myf5^nlacZ/+^* male mouse. At least 20 myofibers per muscle were fixed in 4% paraformaldehyde at T0 (time of isolation) and equivalent numbers were kept in plating medium (10% horse serum (Gibco), DMEM (Gibco), 0.5% chick embryo extract, 4 mM L-glutamine (Sigma), 100 units/ml penicillin and 100 μg/ml streptomycin (Sigma)) for 24 h (T24). After fixation, myofibers were permeabilized with 0.5% Triton X-100 (Sigma), blocked with 10% goat serum and incubated overnight at 4 °C with primary antibodies: Pax7 (DHSB, mouse monoclonal), MyoD (Santa Cruz, rabbit polyclonal), MyoD (Mouse monoclonal, DAKO). Myofibers were then washed in PBS and incubated with the appropriate Alexa-Fluor secondary antibody. Nuclei were counterstained with DAPI (Sigma) (Moyle and Zammit 2014).

### Statistical analysis

Results are expressed as mean ± S.E.M. from the number of samples detailed in the figure legends. Depending on the number of groups compared, one way ANOVA or Mann–Whitney test was used for statistical analysis.

### Microscopy

Fluorescence and bright-field microscopy image were captured using a Zeiss Axiophot (Carl Zeiss, UK, http://www.zeiss. co.uk) microscope and Metamorph (Metamorph Productions, UK, http://metamorphproductions. co.uk) software. Macroscopic pictures of whole X-gal stained muscles were captured with a Leica stereomicroscope (Leica, UK, www.leica-microsystems.com).

Images were assembled into figure panels and minor adjustments to contrast and brightness were made using Adobe Photoshop CS2 (Adobe Photoshop UK, http://www.Adobe.com).

## Results

### *mdx*-derived satellite cells contribute to muscle regeneration

To investigate the contribution of satellite cells to muscle regeneration derived from dystrophin-deficient *mdx* mice, we grafted them into *mdx* nude hosts. As muscle fibers of *mdx* donor origin could not be identified by dystrophin expression, *mdx* mice were bred with 3F-*nlacZ*-2E transgenic mice, whose myonuclei uniformly express β-galactosidase *in vitro* (Beauchamp et al, 2000). This marker is effective for identifying a cluster of dystrophin + ve myofibers as being of donor, rather than of host “revertant” origin in transplantation experiments. However, it will underestimate fibers of donor origin, as when donor satellite cells derived from non-dystrophic 3F-*nlacZ*-2E transgenic mice are transplanted, not all myofibers expressing dystrophin within a transverse cryosection of grafted muscle may contain a β-gal +ve nucleus (Boldrin et al, 2012).

**Figure 1:**
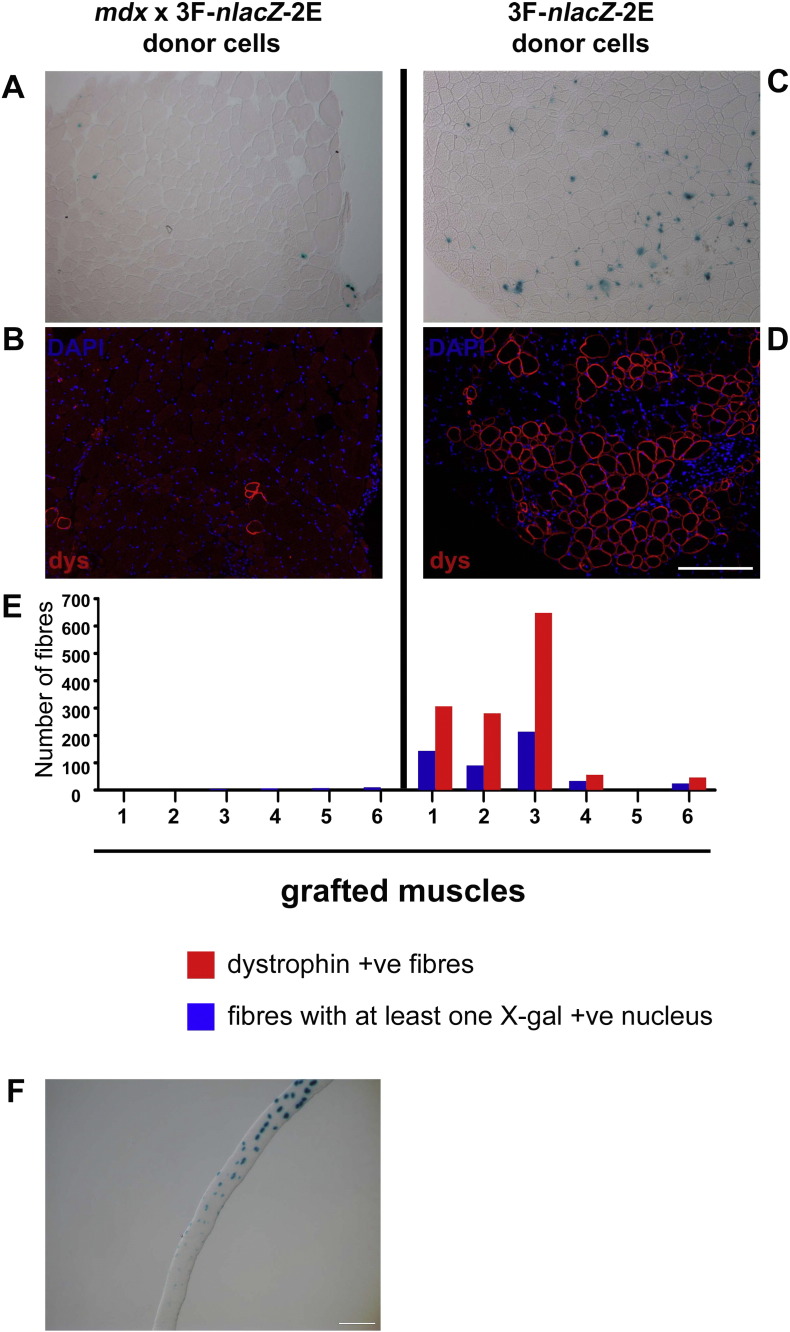
A –D: Donor-derived muscle formation in host mice (*n* = 6), whose right TA muscles were grafted with ~ 400 *mdx* × 3F-*nlaZ*-2E donor satellite cells (donors cells pooled from 3 × 2 month old male mice) and left TA with ~ 400 3F-*nlacZ*-2E (donor cells combined from 3 × age-matched male mice) donor satellite cells. Representative cryosections stained with X-gal (A and C); serial sections stained with dystrophin (dys, red) revealing a few revertant myofibers (B) and several donor-derived myofibers (D) respectively in muscles grafted with *mdx* × 3F-*nlaZ*-2E or 3F-*nlacZ*-2E donor satellite cells. Nuclei in (B) and (D) were counterstained with DAPI. Scale bar = 100 μm. E: Quantification of dystrophin positive myofibers and myofibers containing at least one X-gal +ve nucleus. F: X-gal stained myofiber isolated from *mdx* × 3F-*nlacZ*-2E EDL muscles. Scale bar = 100 μm.

**Figure 2:**
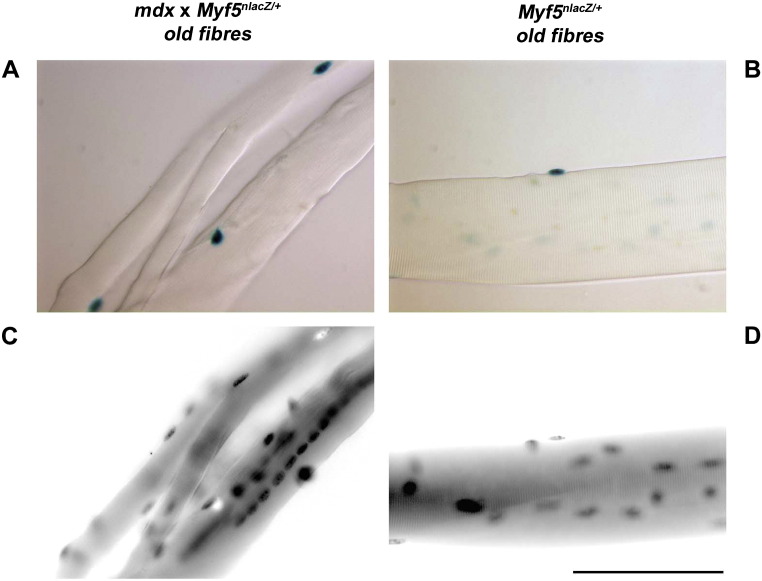
Representative X-gal (A-B) and DAPI (C-D) stained EDL myofibers isolated from *mdx* × *Myf5^nlacZ/+^* (17 month old males, *n* = 3) (A,C) and *Myf5^nlacZ/+^* (B,D) (19 month old male, *n* = 1) mice. *Mdx* × *Myf5^nlacZ/+^* myofibers were all branched (A) and centrally nucleated (C) in all three mice. Scale bar = 50 μm.

Cells isolated from young *mdx* × 3F-*nlacZ*-2E muscles were grafted into TA muscles of *mdx* nude host mice. Surprisingly, very few X-gal +ve nuclei were detected in muscles grafted with *mdx* satellite cells (4.5 ± 1.6 myofibers with at least one X-gal +ve nucleus), indicating significantly less donor-derived myofibers (*P* < 0.05) than those obtained from donor wild-type 3F-*nlacZ*-2E satellite cells (84 ± 33 myofibers with at least one X-gal +ve nucleus and 223 ± 100 dystrophin + ve myofibers) (Fig. 1A–E). It should be noted that the rare, dystrophin +ve, X-gal -ve myofibers (Fig. 2B) in these muscles grafted with donor cells derived from *mdx* mice are most likely host, revertant myofibers (Hoffman et al, 1990; Lu et al, 2000; Yokota et al, 2006).

To test the validity of the donor 3F-*nlacZ*-2E transgene as a marker of muscle in *mdx*, *mdx* × 3F-*nlacZ*-2E isolated EDL myofibers of donor muscles were incubated in X-gal to reveal β-galactosidase activity. This analysis revealed that not all the myonuclei in an isolated myofiber expressed 3F-*nlacZ*-2E (Fig. 1F), suggesting that this marker is not suitable for quantifying muscle regeneration following grafting of *mdx* satellite cells. It is possible that the 3F-*nlacZ*-2E transgene had become inhibited or inactivated during the *in vivo* cycles of muscle degeneration and regeneration in the donor muscle prior to its use to prepare donor cells for transplantation.

### Satellite cells are more numerous in aged *mdx* than in aged wild-type mice and retain their ability to activate

Old *mdx* mouse muscles have undergone several rounds of degeneration and regeneration (Pastoret and Sebille, 1995), thus providing a good model to determine whether the regenerative potential of their satellite cells is compromised as a result.

We first examined the extent of regeneration and hence satellite cell recruitment, in old *mdx* mice. Single fiber analyses and X-gal/DAPI staining revealed that all EDL myofibers derived from old donor *mdx* × *Myf5^nlacZ/+^* were regenerated, as 100% of them were branched (Fig. 2A) and contained regions that were centrally-nucleated (Fig. 2C). By contrast, myofibers from age-matched wild type mice were unbranched, with peripherally located myonuclei (Fig. 2B and D). These branched *mdx* fibers (Fig. 2A) bore 18 ± 2 satellite cells per *mdx* × *Myf5^nlacZ/+^* myofiber. We have previously shown that old *Myf5^nLacZ/+^* mice (19–22 months) have 4.4 ± 0.3 Pax7 +ve satellite cells per EDL myofiber (Boldrin et al, 2009). We analyzed a fourth old (19 month) *Myf5^nlacZ/+^* mouse here and found a comparable number (4 ± 1) of satellite cells per myofiber (Table 1). Combining these current data with the aged *Myf5^nlacZ/+^* mice in (Boldrin et al 2009), shows that aged *mdx* mice have more satellite cells per myofiber than their wild type counterparts (*p* < 0.0001).

Only a minority of *mdx* satellite cells expressed MyoD protein at T0, similar to wild type cells (3% and 7% respectively), indicating that the majority of *mdx* satellite cells was quiescent. When cultured under conditions designed to activate fiber-associated satellite cells (Zammit et al, 2004), we observed that all *mdx*-derived satellite cells retained their capability to activate similarly to wild type cells, with 100% of them expressing Pax7 and MyoD after 24 h (Table 1). These data indicate that the activation status of *mdx* and wild type satellite cells was similar at the time of transplantation.

### Transplanted *mdx* satellite cells reconstitute the satellite cell pool comparably to wild-type satellite cells

**Figure 3:**
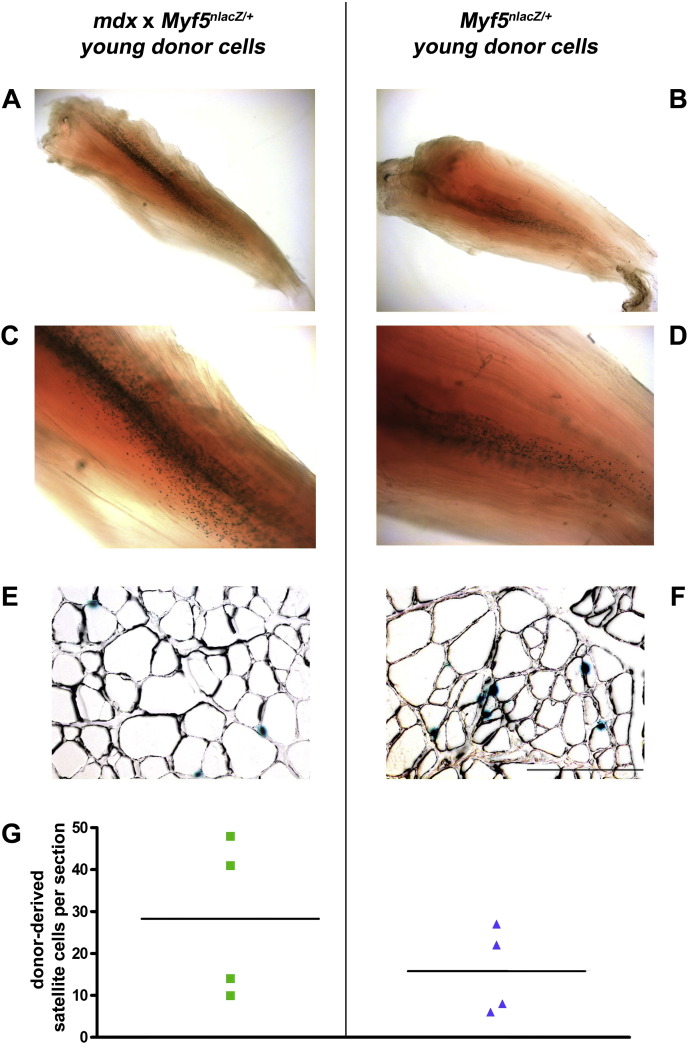
A–D: Representative images of X-gal stained whole muscles (*n* = 8) grafted with either ~ 400 *mdx* × *Myf5^nlacZ/+^* (isolated from a 2 month old male mouse) (A, magnified in C) or ~ 400 *Myf5^nlacZ/+^* (isolated from a 2 month old male mouse) donor satellite cells (B, magnified in D). A,B: 2× magnification; C –D: 5× magnification. E, F: X-gal stained nuclei on cryosections immunostained for laminin. Scale bar = 100 μm. G: Numbers of donor-derived satellite cells per section.

As a marker of satellite cells of donor *mdx* origin, we instead used the targeted *Myf5* locus, as *Myf5^nlacZ/+^* identifies the majority of satellite cells, but not myonuclei, in mature myofibers (Beauchamp et al, 2000). Satellite cells isolated from young *mdx* × *Myf5^nlacZ/+^* muscle gave rise to satellite cells expressing the donor satellite cell marker *Myf5^nlacZ/+^* 4 weeks after transplantation into TA muscles of *mdx* nude recipient mice, as shown by X-gal staining to reveal β-galactosidase activity (Fig. 3A, C). Whole muscle preparations had many satellite cells of donor origin throughout the muscle (Fig. 3A, C), indicating that young transplanted *mdx* × *Myf5^nlacZ/+^* satellite cells gave rise to satellite cells to a similar extent as wild type transplanted satellite cells derived from sex and age-matched *Myf5^nlacZ/+^* donors (Fig. 3B, D).

Quantification of the number of X-gal positive nuclei underneath the basal lamina of myofibers in representative transverse sections (Fig. 3E and F) confirmed that there was no significant difference between the number of *Myf5^nlacZ/+^* satellite cells derived from *mdx* or wild type donor mice (28 ± 10 and 16 ± 5 respectively) (Fig. 3G).

Although *Myf5^nlacZ/+^* is transiently expressed in the centrally-located myonuclei of recently-regenerated mouse muscle fibers (Collins et al, 2005), we analyzed our grafted muscles 4 weeks after cell transplantation, when repair/regeneration derived from donor cells would be completed. Cells expressing *Myf5^nlacZ/+^* within these grafts are therefore predominantly, if not exclusively, satellite cells of donor origin. This was confirmed by the combination of X-gal and laminin immunostaining, showing that the *Myf5^nlacZ/+^* cells are in the satellite cell position, i.e. beneath the basal lamina and at the periphery of the myofiber (Fig. 3E and F) rather than in the center of the myofiber, the characteristic location of a myonucleus in a regenerated myofiber.

### Satellite cells derived from aged *mdx* muscles are as regenerative *in vivo* as their wild type counterparts

**Figure 4:**
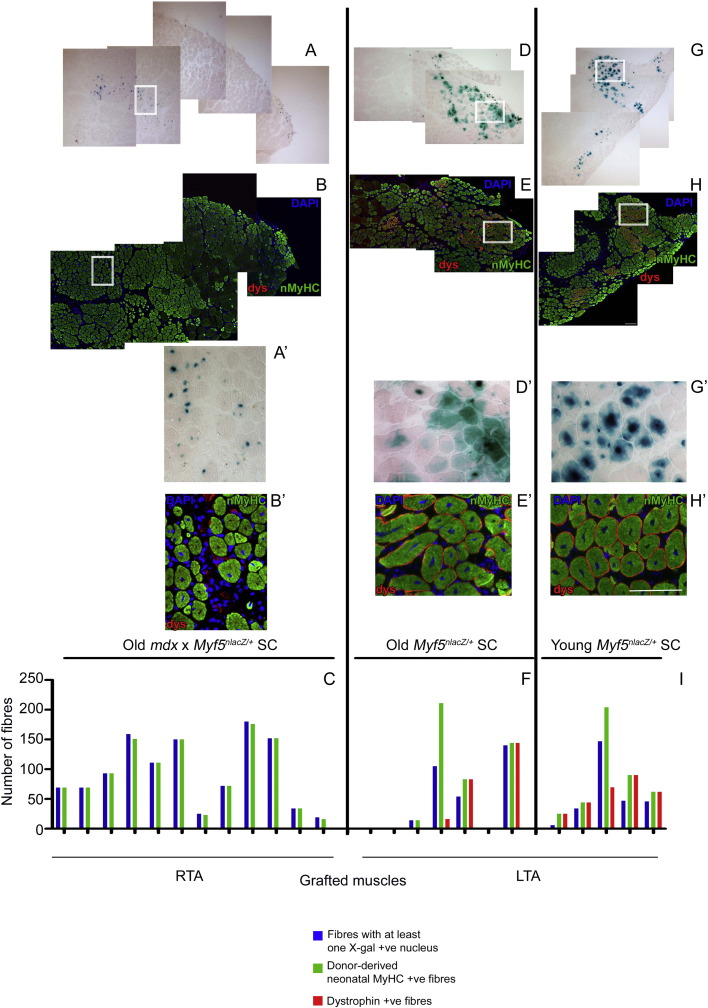
Representative transverse sections from 12 mice (1-12), whose right TA muscles were grafted with ~ 400 satellite cells derived from old *mdx* × *Myf5^nlacZ/+^* mice (donor cells pooled from 2 × 15 month old males) (A, A′, B, B′). Of the same 12 mice, 7 left TA muscles (1-7) were engrafted with ~ 400 old *Myf5^nlacZ/+^* (15 month old male, *n* = 1) (D, D′, E, E′) and the remaining 5 left TA (8-12) muscles were engrafted with ~ 400 young *mdx* × *Myf5^nlacZ/+^* (3 month old male, *n* = 1) (G, G′, H, H′) mice. Sections were either stained with X-gal (A A′, D, D′,G, G′) or immunostained for neonatal MyHC (nMyHC, green) and dystrophin (dys, red) (B, B′, E, E′, H, H′). Boxed area in A,B, D, E, G and H shown at higher magnification in A', B', D', E', G', H'. Scale bar = 100 μm. (C, F, I) depict numbers of myofibers with at least one X-gal + ve nucleus (blue, of donor origin) and neonatal MyHC (green) (i.e. myofibers of donor origin and newly regenerated). (F, I) Where Myf5nlacZ/+ were transplanted, dystrophin was also quantified. Nuclei in B, E, H and B′, E′, H′ were counterstained with DAPI.

To explore the *in vivo* functionality of satellite cells derived from aged *mdx* × *Myf5^nlacZ/+^* mice compared to aged (or young) *Myf5^nlacZ/+^* mice, satellite cells were grafted into TA muscles of recipient *mdx* nude mice. Three weeks after transplantation, regenerated muscles were injected with notexin, which destroys myofibers, but spares satellite cells (Harris, 2003), to test whether donor-derived cells had retained the ability to contribute to muscle regeneration. Muscles were analyzed 7 days later by X-gal staining and immunostaining, as this is the time at which mouse myofibers that have regenerated in response to notexin are expressing neonatal myosin heavy chain (MyHC) (Gross and Morgan, 1999). Neonatal MyHC marks newly-regenerated, but not mature, myofibers, permitting ready quantification of the notexin-induced regenerative response. In addition, myonuclei of donor origin will still express *Myf5^nlacZ/+^* a week after the fiber had begun to regenerate (Collins et al, 2005), as this becomes down-regulated only after fiber maturation. Thus, a newly-regenerated myofiber of donor origin expressing neonatal MyHC, whose myonuclei contain β-galactosidase, is evidence that satellite cells of donor origin are functional (Boldrin et al, 2012; Boldrin et al, 2009). We found no difference in the number of newly regenerated myofibers of donor origin (Fig. 4A–I) derived from aged *mdx* × *Myf5^nlacZ/+^* satellite cells (93 ± 16) compared to young (85 ± 32) or aged *Myf5^nlacZ/+^* (65 ± 32) satellite cells.

As confirmation of the utility of *Myf5^nlacZ/+^* as a marker of myonuclei in newly-regenerated myofibers of donor origin, we used dystrophin as a second marker of muscle fibers of wild-type donor origin. Analysis revealed that all dystrophin positive myofibers were newly regenerated as shown by neonatal MyHC immunostaining (Fig. 4F and I).

## Discussion

Duchenne muscular dystrophy (DMD) is a chronic and debilitating genetic disorder in which muscle regeneration fails to compensate for the loss of muscle tissue (Emery, 2002).

It has been suggested that inadequate muscle regeneration in muscular dystrophies may be due to loss of satellite cells, which after many rounds of muscle degeneration and regeneration, become ‘exhausted’ (Morgan and Zammit, 2010). In particular, in *mdx* muscles, the myogenic activity of satellite cells has been reported to be lost with age (Smythe et al, 2008) and the “stem cell” fraction of satellite cells appears to be depleted (Heslop et al, 2000). However, in contrast to those findings, we show that satellite cells isolated from *mdx* mouse muscles have a similar regenerative capacity and ability to give rise to functional satellite cells as wild type satellite cells, when transplanted into a permissive host muscle environment.

In contrast to DMD patients, skeletal muscles of *mdx* mice retain their ability to regenerate throughout life. Muscle fiber degeneration/regeneration peaks at 3 weeks of age and continues throughout life, albeit with less intensity (Muntoni et al, 1993; Pastoret and Sebille, 1995). Evidence of ongoing muscle degeneration and regeneration can be found in old *mdx* mouse muscles (Chamberlain et al, 2007), with myofibers progressively lost and pathological features becoming more severe with age (Blaivas and Carlson, 1991; Pastoret and Sebille, 1993; Pastoret and Sebille, 1995; Pichavant and Pavlath, 2014). It was therefore intriguing to explore the regenerative potential of satellite cells derived from old, rather than young, *mdx* mice, as it could be hypothesised that their capacity to regenerate skeletal muscle would decrease with increasing age. Surprisingly, once transplanted into muscles of young host mice, the contribution of satellite cells derived from aged *mdx* donors to muscle regeneration, as a result of the combination of their engraftment and response to widespread myofiber destruction (notexin injection), was comparable to old and young wild type satellite cells.

We also showed that in the old *mdx* × *Myf5^nlacZ/+^* mouse, whose muscle pathology is exacerbated by age, satellite cell number per myofiber is not reduced compared to their wild type counterparts (Boldrin et al, 2009). In fact, there were more than 4 fold more satellite cells per myofiber, and although old *mdx* myofibers are branched, they do not have 4 fold the volume of equivalent wild type ones. This is in contrast to a recent paper that showed that satellite cell number/EDL myofiber was elevated in 6 month old C57Bl/10 *mdx* mice (approximately 18 Pax7 + cells/fiber) and reduced thereafter (approximately 5 cells/fiber at 24 months of age) (Jiang et al, 2014). This difference could be due to the genetic background and/or on the sex of the mice analyzed, as male and female adult mouse EDL myofibers bear different number of satellite cells (Neal et al, 2012). However, satellite cell number is also elevated in DMD patients relative to controls (Bankole et al, 2013; Kottlors and Kirschner, 2010). Unlike our findings in *mdx*, reduction of satellite cell number is a hallmark of some other mouse models of muscular dystrophies, e.g. the *lmna*^−/−^ mouse, whose satellite cell content is decreased compared to wild type mice (Gnocchi et al, 2011).

The vast majority of satellite cells from old *mdx* mice did not express MyoD, indicating that they are quiescent, as found with wild type satellite cells. The Large^myd^ mouse model of dystroglycanopathy, in which there are defects in the glycosylation of α-dystroglycan (a component of the dystrophin-associated glycoprotein complex) (Muntoni et al, 2004), has significantly more satellite cells than wild type control muscles, at 2–3 months of age (Ross et al, 2012). Unlike *mdx*, however, significantly more satellite cells in Large^myd^ mouse muscles are activated (expressing MyoD) or differentiating (expressing myogenin) (Ross et al, 2012). Earlier differentiation has also been observed for *mdx* satellite cells (from 8 to 11 week old mice) expanded *in vitro*, compared to satellite cells derived from control, wild type mice (Yablonka-Reuveni and Anderson, 2006).

Factors within the host dystrophic environment that may be detrimental to effective muscle regeneration include the inflammatory milieu and/or fibrotic environment (Abou-Khalil et al, 2010; Boldrin et al, 2009; Emery, 2002; Mann et al, 2011). For effective donor-derived muscle regeneration, the host muscle environment has to be modulated (Boldrin et al, 2012; Boldrin et al, 2009; Brimah et al, 2004; Morgan et al, 2002). In the *in vivo* model that we use to assay donor satellite cell function, the recipient muscles had been pre-irradiated in order to create an environment permissive to donor-derived muscle regeneration (Boldrin et al, 2009; Collins et al, 2005), by reducing competition with endogenous satellite cells and preserving the host satellite cell niche (Boldrin et al, 2012). Modifications of the host muscle environment are indeed critical to permit effective muscle regeneration (Boldrin et al, 2009; Collins et al, 2005). For example, it has recently been shown that miR-29 expression is down-regulated in muscles of *mdx* mice; by restoring its expression, the dystrophic pathology improves, as regeneration is promoted and fibrogenesis inhibited (Wang et al, 2012).

In summary, we found that satellite cells from *mdx* mice retain their capacity to contribute to muscle regeneration within pre-irradiated muscles of *mdx* nude host mice. This is evidence that, when removed from the pathological donor muscle environment and placed within a permissive milieu, satellite cells retain their muscle stem cell properties and contribute effectively to muscle regeneration. Therefore, we conclude that the dystrophic muscle environment is deleteriously affecting satellite cell-derived muscle regeneration. This suggests that dystrophic muscle could be modified to improve either endogenous muscle regeneration, or for therapy involving engraftment of stem cells.

## Figures and Tables

**Table 1:** Number of quiescent (Pax7 +) and activated (Pax7 +/MyoD +) satellite cells on myofibers isolated from *mdx* × Myf5*^nlacZ/+^* (17 month old males, *n* = 3) and Myf5nlacZ/+ (19 month old male, *n* = 1) EDL muscles. Percentages of fibers with satellite cells expressing Pax7 or Pax7 and MyoD are also presented. At least 20 fibers were analyzed from each mouse at T0 and T24.

	Pax7	Pax7/MyoD
*Old Myf5^nlacZ/+^*		
T0	3.9 (± 0.5) 93%	0.3 (± 0.15) 7%
T24	0	3.2 (± 0.29) 100%

*Old mdx X Myf5^nlacZ/+^*		
T0	17.46 (± 1.47) 97%	0.5 (± 0.16) 3%
T24	0	14.56 (± 1.48) 100%
